# Ammonia Volatilization and Greenhouse Gases Emissions during Vermicomposting with Animal Manures and Biochar to Enhance Sustainability

**DOI:** 10.3390/ijerph18010178

**Published:** 2020-12-29

**Authors:** Syed Turab Raza, Jia Liang Tang, Zulfiqar Ali, Zhiyuan Yao, Hamidou Bah, Hassan Iqbal, Xiao Ren

**Affiliations:** 1 Key Laboratory of Mountain Surface Processes and Ecological Regulation, Institute of Mountain Hazards and Environment, Chinese Academy of Sciences, No. 9, Section 4, Renmin Road-South, Chengdu 610041, China; s.turabkazmi@hotmail.co (S.T.R.); yaozhiyuan298@foxmail.com (Z.Y.); bahamidou2004@gmail.com (H.B.); renxiao@imde.ac.cn (X.R.); 2 International College, University of Chinese Academy of Sciences, Beijing 100049, China; 3 Laboratory of Environmental Health & Wildlife, Department of Zoology, University of the Punjab, Lahore 54590, Pakistan; zali.zool@pu.edu.pk; 4 Xinjiang Institute of Ecology and Geography, Chinese Academy of Sciences, Urumqi 100864, China; hassancp1477@mails.ucas.edu.cn

**Keywords:** vermicomposting, *Eisenia fetida*, nutrient cycling, C/N C/P ratios, maize residues, cumulative loadings

## Abstract

There is a huge potential for nutrient recovery from organic waste materials for soil fertility restoration as well as negative environmental emission mitigation. Previous research has found vermicomposting the optimal choice for converting organic waste into beneficial organic fertilizer while reducing reactive N loss. However, a great deal of the processes of greenhouse gases (GHG) and ammonia volatilization during vermicomposting are not well-documented. A field vermicomposting experiment was conducted by deploying earthworms (*Eisenia fetida*) with three types of agricultural by-products—namely, cow manure (VCM), pig manure (VPM), and biochar (VBC)—and crop (maize) residues compared with traditional composting (COM) without earthworms in the Sichuan Basin, China. Results showed that vermicomposting caused a decrease in electrical conductivity (EC) and total organic carbon (TOC) while increasing total nitrogen (TN). The greatest TN increase was found with VCM. The cumulative NH_3_ volatilization in COM, VCM, VPM, and VBC during experimental duration was 9.00, 8.02, 15.16, and 8.91 kg N ha^−1^, respectively. The cumulative CO_2_ emissions in COM, VCM, VPM, and VBC were 2369, 2814, 3435, and 2984 (g·C·m^−2^), while for CH_4_, they were 0.36, 0.28, 4.07, and 0.19 (g·C·m^−2^) and, for N_2_O, they were 0.12, 0.06, 0.76, and 0.04 (g·N m^−2^), respectively. Lower emissions of N_2_O, CH_4_, and NH_3_ were observed in VBC. We concluded that earthworms, as ecological engineers, enhanced reactive nutrients and reduced ammonia volatilization during vermicomposting in our test system. Overall, vermicomposting is proposed as an eco-friendly, sustainable technique that helps to reduce environmental impacts and associated health risks.

## 1. Introduction

Vermicomposting is a method used to produce compost by employing earthworms. It converts organic matter into high-quality compost along with decomposed organic nutrients [1]. Vermicompost is small, peat-like, finely divided materials with high water-holding capacity, aeration through microbial activities, porosity, and drainage [2]. These composts are formed through interactions of microorganisms and earthworms, causing non-thermophilic stabilization and the degradation of organic material [3]. Vermicompost is regarded as an ecological alternative to recycling organic waste material. It also acts as a soil conditioner that helps plant growth [4]. Organic wastes that can be used for vermicomposting for positive influence of plant growth [5] include crop remnants, animal dung, sewage sludge, and discarded industrial materials [5,6]. The use of renewables is rising rapidly, especially in developed countries, which is complemented by more intense use of agrochemicals and additional agrotechnical operations such as biochar addition. However, the increased cost is beginning to threaten the economic sustainability [7].

According to estimates, about 2.6 million tons per day (TPD) of municipal solid waste is produced globally, and the amount may reach up to 4.5 million TPD by 2050 [8]. Similar to other countries, China has serious environmental concerns as a result of solid waste [9] and organic leftover raw materials. A total of 630 billion tons of crop residues [10] and 219 million tons of pig manure [11] are produced each year, necessitating a method of recycling those wastes to prevent potential agro-environmental pollution. Recycling organic waste through composting and vermicomposting has gotten serious consideration for a number of years [12]. Composting through aerobic pathways has gained attention because they can transform solid wastes into nutrient-rich materials [13] while maintaining a low cost, high nutrient-producing capacity [14]. Furthermore, vermicompost with solid municipal waste materials can create high profit margins [15] with less ecologically harmful effects than other ways of managing waste materials, i.e., incineration and landfilling. *Eisenia fetida* is one of the most important members of the family Lumbricidae, which is used for vermicomposting [16]. The individuals of Lumbricida were naturally the most found, dominant group in soil fauna of purplish soil, southwest of China, that can be used for vermicomposting [17].

Biochar is solid carbon produced by the thermal conversion of organic waste materials, including plant biomass and algal substances, and it can be produced under anaerobic or low-oxygen conditions [18]. In recent years, biochar has also been used as a soil ameliorator. It activates other materials used for composting that have horticultural and ecological advantages, including microbial biomass. For this purpose, biochar could be mixed with manure for nutrient recycling during composting [19] and has been recognized as a potential vermicomposting material in some research [20]. Vermicomposting technology for nutrient cycling has already been applied in a subtropical upland region [21] using manure and biochar with crop residues. In this study, biochar was used for promoting earthworm growth and vermicompost quality [22]. Biochar is also useful for wastewater treatment [23], but its use in commercial agriculture remains scarce [24]. Several aspects to the usage of biochar substrates may impact further planning strategies [25], especially on role of agriculture in economy [26]. Various researchers have studied the basic economic analysis [27], and the local social impacts for sustainability of agriculture entities [28].

However, the real effects of biochar during vermicomposting for potential agricultural applications have not been comprehensively elucidated.

Much research has dealt with the environmental effects of vermicomposting [27], especially related to greenhouse gas emission. A few studies have been conducted on NH_3_ volatilization [28] and greenhouse gas emission simultaneously, specifically for vermicomposting amended with biochar [29]. NH_3_ volatilization is considered an important pathway for nitrogen loss [25] during composting [29], which could be harmful because of its contribution to acid aerosols. Greenhouse gases (GHG) and their cumulative emissions from combined pre-composting and vermicomposting of duck manure was comprised of N_2_O (92.8–274.2), CH_4_ (5.8–30.4), and CO_2_ (260.6–314.0) mg/kg DM. It is shown that earthworms and amendments significantly decreased N_2_O and CH_4_ emissions [30,31]. Researches have also focused to study the mechanisms that how earthworms affect GHG emissions in soil [32], but less information is available regarding their loadings [33]. Nutrient cycling with the emission of gases has been studied with different composting substrates; most of them were lab based small-scale experiments and did not take into account winter conditions that can exist in the field. A comprehensive field-scale study following NH_3_ volatilization, GHG emissions, and nutrient status during vermicomposting with manures and biochar is needed to fill in the gaps in the information currently available.

Our goal is to recover plant nutrients from waste materials while lowering environmental loadings by using eco-friendly techniques. The objectives of the study were to (1) monitor nutrient dynamics during the decomposition of organic wastes with and without earthworms in the field; (2) quantify the NH_3_ volatilization rates and GHGs emission fluxes during vermicomposting; and (3) identify effective vermicomposting substrates by comparing maize crop residues amended with cow manure, pig manure, or biochar. Crop residues with cow manure and no added earthworms were used as a control. 

## 2. Materials and Methods

### 2.1. Description of the Study Site

**Figure 1:**
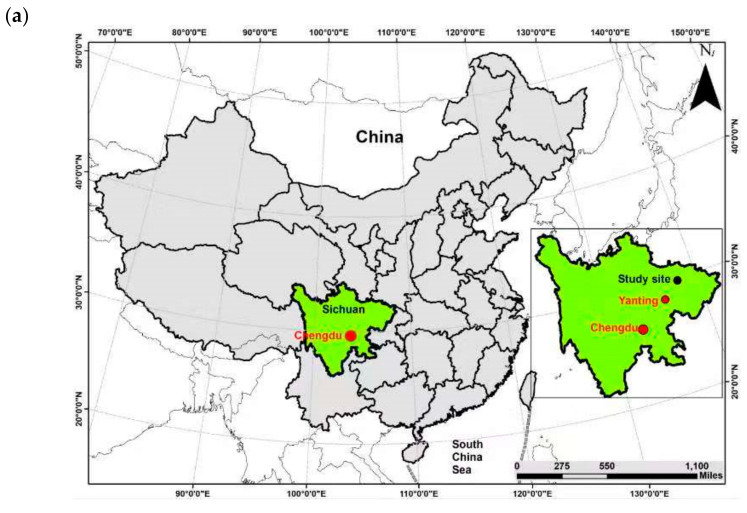

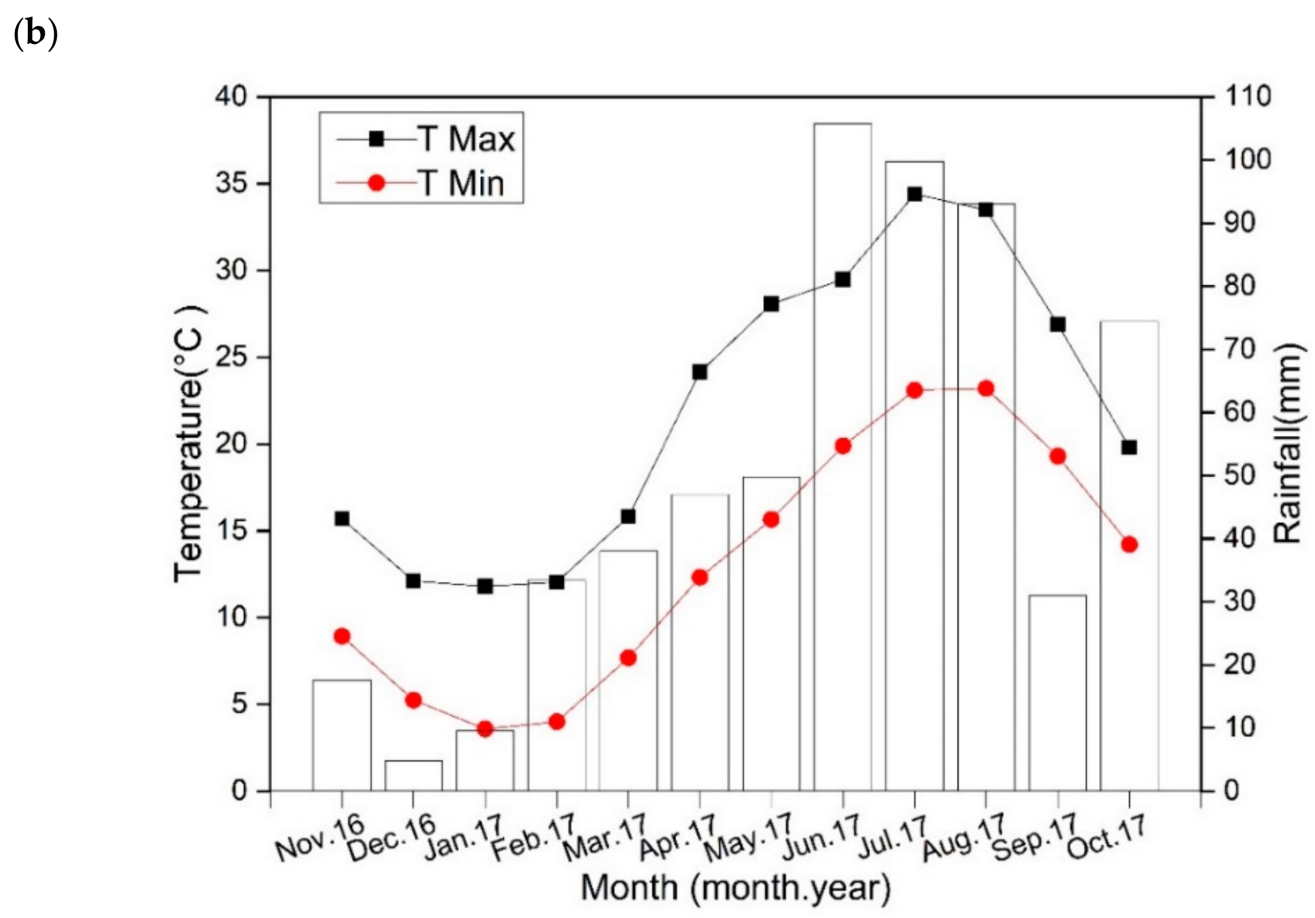
(**a**) Location of the study site, (**b**) climatic conditions during the preparation of vermicompost November 2016–October 2017.

A field vermicomposting experiment was established at Yanting Agro-ecological experimental station, which is a member of the Chinese Ecosystem Research Network (CERN), Chinese Academy of Sciences, located 31°16′ N, 105°28′ E at an altitude of 530 m, in Sichuan Basin. The area’s climatic conditions are subtropical monsoons, and the average annual temperature is 17.3 °C, while the average annual precipitation is 836 mm [33]. The location of the study site and the climatic conditions along with monthly maximum and minimum temperature with rainfall during the experimental period are shown in Figure 1.

### 2.2. Material Collection for Vermicomposting

Maize residues, cow manure, and pig manure were collected from nearby breeding farms. An earthworm species *Eisenia fetida* was bought from the market and used for vermicomposting, while commercial biochar was purchased from Sanli New Energy Company at Shangqiu, Henan, China. Biochar was produced from one ton of crop straws by slow pyrolysis at 500 °C in a fluidized bed furnace on a scale of 1000 t·day^−1^ [34]. One ton of crop straw produced 0.3 t biochar, with 0.25 t pyroligneous acid, 0.03 t wood tar, and 780 m^3^ gases as coproducts. The physicochemical properties of all raw materials used during the experiment are listed in Table 1.

### 2.3. Experimental Design of Vermicompost

The twelve plots were designed with dimensions 2 m (l) × 1.5 m (w) × 0.6 m (h) for each. Four treatments with three replicates each were used. Plots were amended with 200 kg of one of the following: compost (COM): crop residues 70% by mass, cow manure 30%, no earthworms; vermicompost (VCM): crop residues 70%, cow manure 30%, + earthworms: vermicomposting with pig manure (VPM): crop residues 70%, with pig manure 30%, + earthworms; vermicomposting with biochar (VBC): crop residues 70%, biochar 30%, + earthworms.

The plots were covered by a stainless steel shelter, providing a roof for the plots (to avoid direct sunlight and rainfall). Plots were separated by cemented walls with holes at the lower corner of plot for the maintenance of extra moisture (provided by the sprinkling of water). The plots were first filled with purple soil (“Pup-Orthic Entisols” as per Chinese soil classification; FAO classified as “Eutric Regosol” [35]; specific physicochemical properties described by [12]) up to 15 cm as a base layer. Maize residues (harvested in the previous season) of approximately 10 cm length were added to the plots as a bedding layer. Cow and pig manure were added on a bedding layer in two plots (with replicates), while biochar was used instead of manure in other plots. The basic physicochemical properties of the raw materials are recorded in Table 1.

Five hundred earthworms (*Eisenia fetida*, both with clitellum and non-clitellum) were added to each of the VCM, VPM, and VBC plots. Water was sprinkled to maintain moisture at 60–70% for each plot. Bedding material was sampled at twenty-day intervals from December 2016 to April 2017. The first sample was taken after the initial layering of materials in plots. As the bedding materials in the first weeks were harmful to soil fauna, earthworms were added on day 18, and then the compost was thoroughly mixed again. Then, frequent mixing of compost piles in all treatments with different intervals (about 2–3 weeks) for better aeration was performed during the whole experimental period.

### 2.4. Nutrient Contents and Physicochemical Properties of Vermicompost

A CN elemental analyzer (Elemental Analysen Systeme GmbH, Vario M Cube, Langenselbold, Germany) was used to measure TN and TOC. The wet digestion method was employed to measure the concentration of total phosphorous (TP) using a spectrophotometer [36]. Dry samples of compost material (2 g per replicate) were acidified with 10 mL di-acid (1:5 HClO_4_:HNO_3_) brought to 100 mL with water and filtrated through filter paper (Whatman No.1). A Flame Atomic Spectrophotometer was used to analyze total potassium (TK) concentration in the sample (Thermo Scientific, iCE 3000 SERIES, Cambridge, UK). Fresh samples were collected at each interval of mixing compost piles for nitrate–nitrogen (NO_3_-N), dissolved organic carbon (DOC), and ammonium nitrogen (NH_4_-N). Samples were stored in a refrigerator at 4 °C until analysis using continuous flow-Auto Analyzer (AA3, Bran- Luebbe, Nordersteld, Germany).

Compost samples were analyzed for pH and electrical conductivity (EC) with 1:10 aqueous suspension [30].

### 2.5. Ammonia Volatilization

Ammonia volatilization was measured by small open static dynamic chamber method [37]. The poly-methyl methacrylate chamber was 20 cm (inner diameter) × 10 cm (height). A vacuum pump was used to exchange ambient air at the height of 2.5 m above the compost surface, with a flow rate set at 5–10 min^−1^ through the chamber. The circular chamber was inserted into the compost at a depth of 8–10 cm. NH_3_ was trapped in glass bottles containing 50 mL of 0.01 mol L^−1^ sulfuric acid solution. In the first two weeks, the samples were taken twice a day: in the morning at 9–10 AM and in the afternoon at 4–5 PM. In the third week, samples were taken two times per week. Ammonium nitrate N (NH_4_-N) in the acid trap was titrated with 0.01 mol L^−1^ standard diluted sulfuric acid solution, which was determined calorimetrically with a flow injection auto-analyzer. NH_3_ flux was calculated by the following equation [38],

(1) $$F=\frac{2\left(C\right)\left(V\right)\left(14\right)\left(10^{-2}\right)}{\pi (R^{2})}\times \frac{24}{t}$$

where *F* is the total flux of ammonia volatilization (kg N/ha/d), C is H_2_SO_4_ concentration (mol/L), V is the volume of H_2_SO_4_ consumed (at standard dilution), *t* is the elapsed time (h), and R is the chamber radius (m). For the total NH_3_ losses, the sum of all the values of volatilization fluxes for the whole experimental period was determined.

### 2.6. Greenhouse Gas Emissions

Measurements of CO_2_, N_2_O, and CH_4_ were conducted by static chamber gas chromatography [12]. A stainless-steel chamber (50 × 50 × 50 cm) was introduced into the 10 cm deep compost. The chamber was fully wrapped by an insulating sheet to reduce the chances of temperature changes during gas sampling. Samples were collected daily for the first week, then every two days, and finally twice per week to the end of the experiment. Five samples were taken every 7 min with 60-mL syringes connected to the closed chambers through Teflon tubes with 3-way stopcocks. During gas sampling, the temperature inside the chamber and the compost temperature were also determined by a manual thermocouple thermometer. The collected samples were carried to the lab, where a Gas Chromatograph (HP-5890 Series II, Hewlett- Packard Alto, GC, San Diego, CA, USA) was fitted to an electron capture detector (ECD) for analysis. CO_2_, CH_4_, and N_2_O fluxes were determined from linear or non-linear values as increased, judged on basis of *r*^2^ values, in their headspaces with time, thereby considering chamber headspace height and pressure, and temperature of the air. The method was used to measure the emission of greenhouse gases on sampling days [39]. The cumulative gas emissions were determined by the following equation described by Meng et al. [40].

(2) $$C\;=\;\frac{\sum F_{i+1}+F_{i}}{2}\times \left(t_{i+1}-t_{i}\right)\times \;24\;$$

where *C* represents cumulative emission, *F* is flux, *i* is the initial day of sampling, and *t* represents time (days).

Global warming potential (GWP) was calculated to evaluate the total global warming effects for total GHG emissions. Cumulative CO_2_, CH_4_, and N_2_O emissions were transformed to CO_2_- equivalents and summed to get total GHG discharges as warming potential (GWP; 1 mol CH_4_ = 34 mol CO_2_-equivalent, 1 mol N_2_O = 298 mol CO_2_-equivalent) [31,41,42]. CO_2_ emissions, which are usually treated as anthropogenic in a global context, were also considered biogenic here. 

### 2.7. Statistical Analysis

Microsoft Excel and Origin 2018 were used for data processing and making graphs. Statistical analyses were done using SPSS software 15.0. One-way Analysis of Variance (ANOVA) was applied to study the difference in selected parameters with Tukey’s test. Significant differences between treatments in triplicates were also verified by using the least significant difference (LSD) test. 

## 3. Results

### 3.1. pH and Electrical Conductivity during Vermicomposting 

**Figure 2:**
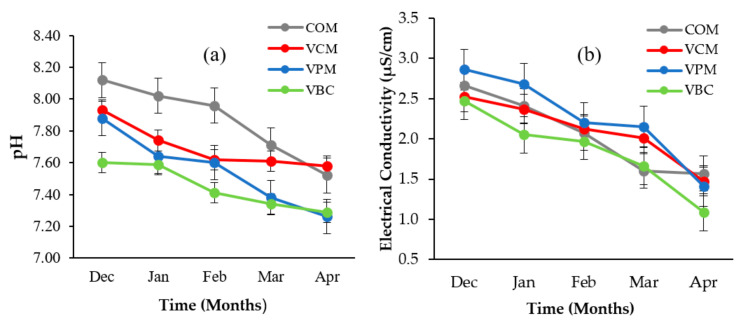
Temporal variations of pH (**a**) and electrical conductivity (**b**) over a period of five months for four treatments. Error bars represent standard errors (*n* = 3).

Variation in pH and EC during vermicomposting for different substrates are provided in Figure 2a,b. pH showed a decreasing trend in all treatments (COM, VCM, VPM, and VBC).

A similar trend was observed by other scientists [31,43] during vermicomposting with livestock dung and crop straw. EC decreased in all treatments but the minimum values were recorded in VBC (1.09 µS/cm).

### 3.2. Nutrient Content Dynamics during Vermicomposting

**Figure 3:**
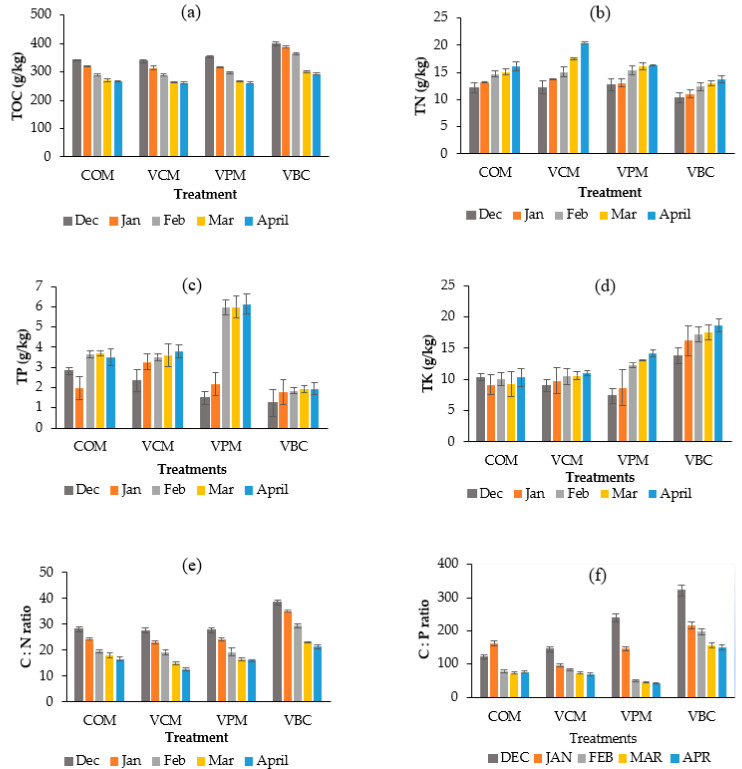
Temporal variations of total organic carbon (**a**), total nitrogen (**b**), total phosphorous (**c**), total potassium (**d**), C:N ratio (**e**), C:P ratio (**f**), over the period of five months of the experimental duration. Error bars represents standard errors (*n* = 3).

The TOC content showed a decreasing trend for all treatments (Table 2, Figure 3a). It decreased significantly more in VPM and VBC than in COM (*p* < 0.05).

TN contents increased for all studied treatments (Figure 3b, Table 2), and the maximum increase was recorded in VCM (65.85%). TN showed significant differences among treatments, while VBC showed significant difference as compared to COM (*p* < 0.05). The lowest was recorded in VBC (31.73%).

TP contents (Figure 3c, Table 2) increased during the observation period in all vermicompost treatments when compared with COM, with the greatest increase seen in VPM (from 1.48 to 6.13 g/kg). A similar increase (1.4 to 9.7 g/kg) was seen for pig slurry by Swarnam et al. [41]. Similar results had been observed with pig slurry and cow manure in other studies The total potassium (TK) contents increase in VBC were 35.56% (Figure 3d, Table 2), and a significant increase was also observed in the VPM treatment, but only slight increases were observed in VCM, and there was no variation in COM (*p* < 0.05). VBC was significantly lower than COM, VCM, and VPM [42,43]. 

The C:N ratio is the key factor in determining compost and vermicompost maturation [44]. Overall, C:N ratios of vermicompost decreased in all treatments (Table 2), particularly in VCM and VBC.

The C:P ratio decreased in all four treatments (Figure 3f), with greater decreases in the three worm treatments than in COM. The N:P ratios generally showed increasing trends in all treatments except in VPM (Table 2), with values around 3–6. 

**Figure 4:**
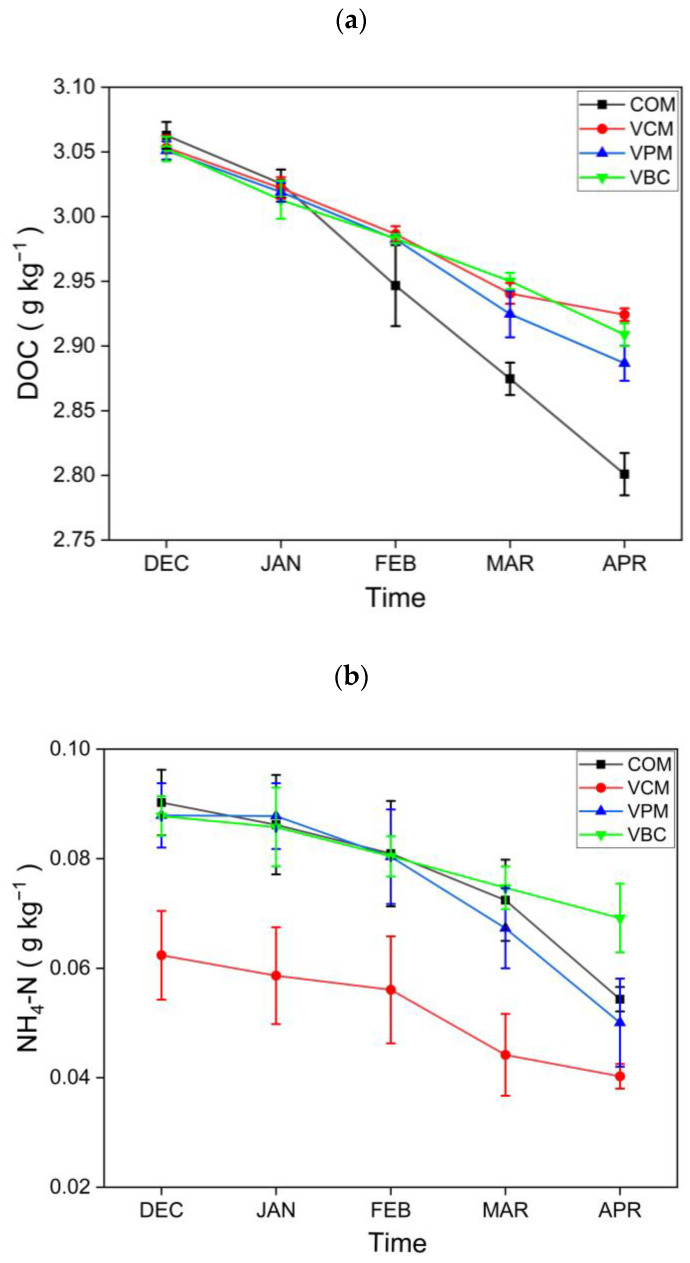

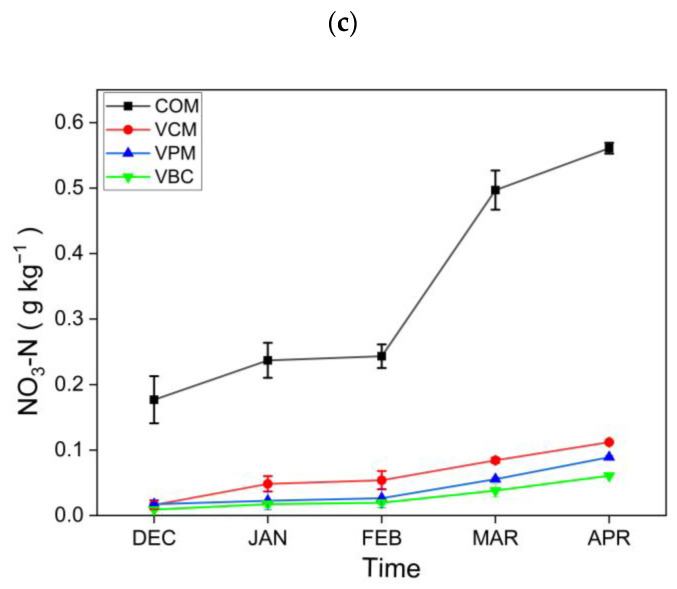
Dissolved organic carbon: (**a**) nitrogen NH_4_^−^N, (**b**) NO_3_^−^N content, (**c**) content in all treatments. Error bars represents standard errors (*n* = 3).

For plant-available nutrients in composts, dissolved organic carbon (DOC) showed a downward trend in all treatments, as shown in Figure 4a. The results for DOC are comparable with [45], who concluded that earthworms alter the amount as well as the composition of organic matter. The NH_4_^+^–N contents (Figure 4b) showed a declining trend in all treatments, while NO_3_^−^–N contents (Figure 4c) were increasing with time during the observation period. Similar trends were also observed in the vermicomposting of cattle dung in a previous study [46]. Earthworms may improve aerobic conditions during the vermicomposting of cow dung, pig manure, and biochar, and they also enhance the nitrification process, as suggested by higher NO_3_-N for the three vermicomposting treatments.

### 3.3. NH_3_ Volatilization during Vermicomposting

**Figure 5:**
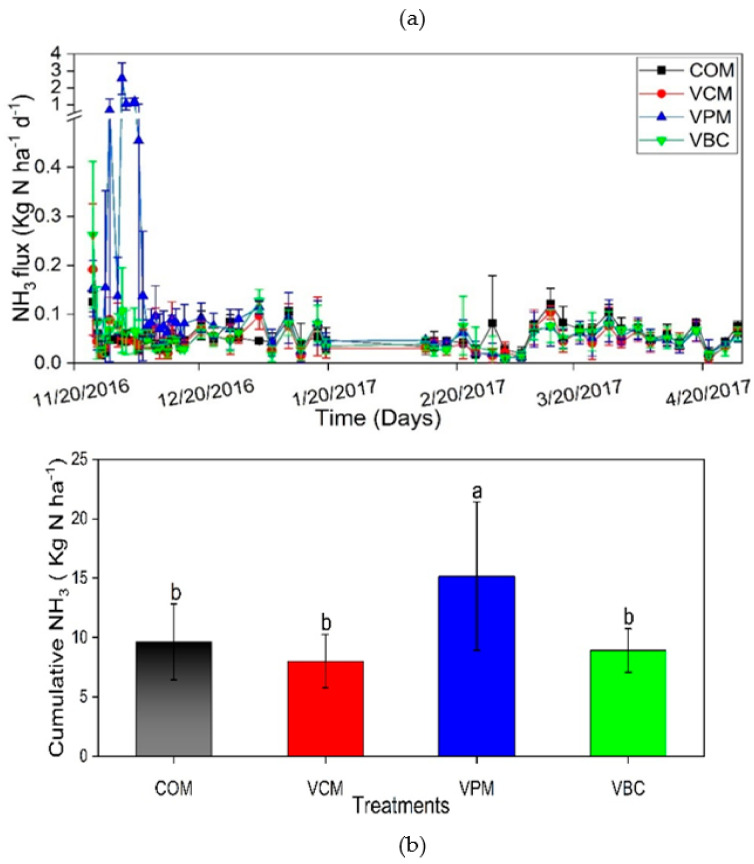
NH_3_ volatilization (**a**), cumulative NH_3_ (**b**), during experimental duration. Error bars represent standard errors (*n* = 3). Different letter indicates significant differences among treatments (*p* < 0.05).

Ammonia emissions peaked during the pre-composting period in all treatments (Figure 5a). When mixed with water, VPM showed the maximum NH_3_ followed by VBC, VCM, and COM with values 0.14, 0.08, 0.08, and 0.05 kg N/ha d, respectively. 

The cumulative value of NH_3_ volatilization of VPM was about 15 kg N/ha, which was significantly higher than the other three treatments (*p* < 0.01) (Figure 5b). Most NH_3_ was emitted within the first week before the compost was mixed with water, which is consistent with earlier observations [22].

### 3.4. Greenhouse Gas (CO_2_, CH_4_, and N_2_O) Emissions during Vermicomposting

**Figure 6:**
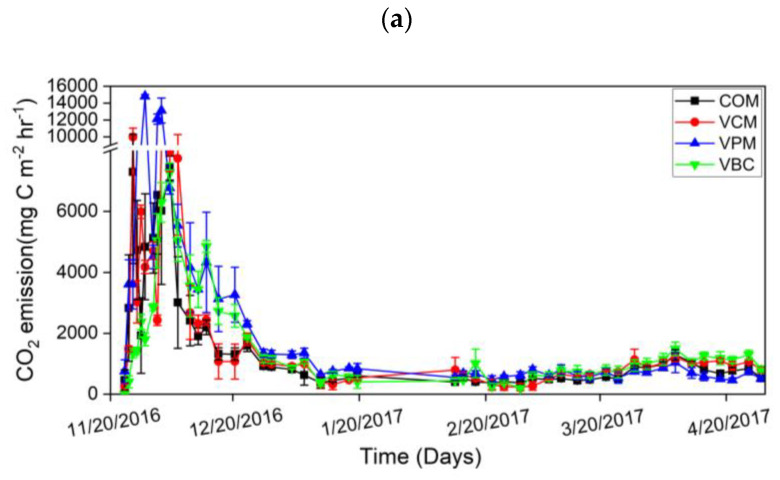

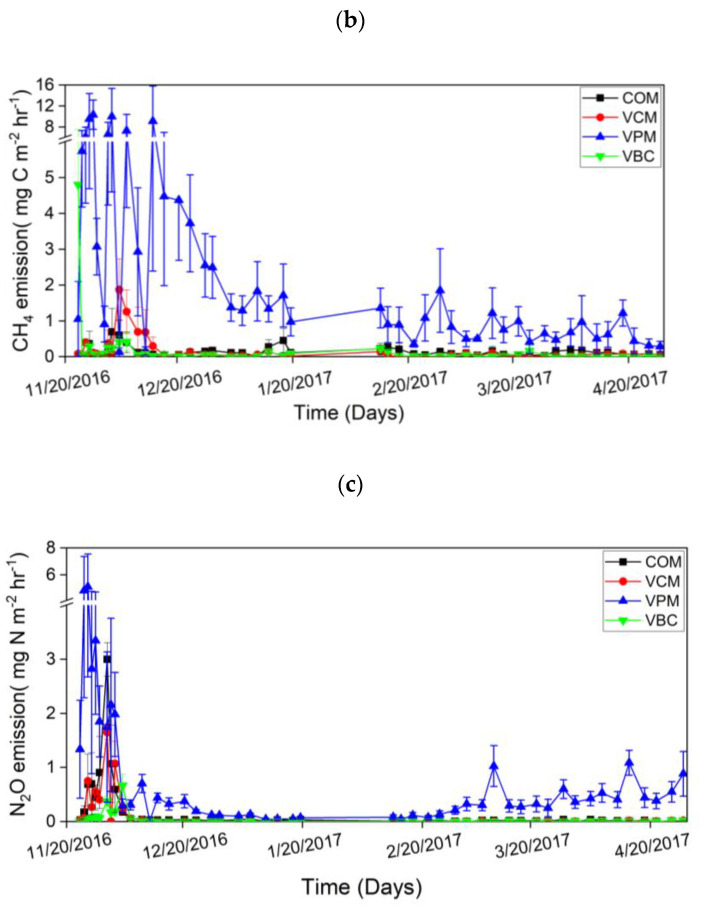
Carbon dioxide emission (**a**), methane emission (**b**), nitrous oxide emission (**c**), during experimental duration. Error bars represent standard errors (*n* = 3).

In the first week, all treatments resulted in high CO_2_ emissions (Figure 6a). Afterward, with the addition of water in the second week in all compost piles, CO_2_ emissions reached their highest peaks and then showed a downward trend over the experimental duration. VPM exhibited maximum variation during the pre-composting period. It is shown that earthworms can hasten the decomposition of organic matter and increase CO_2_ emissions.

**Figure 7:**
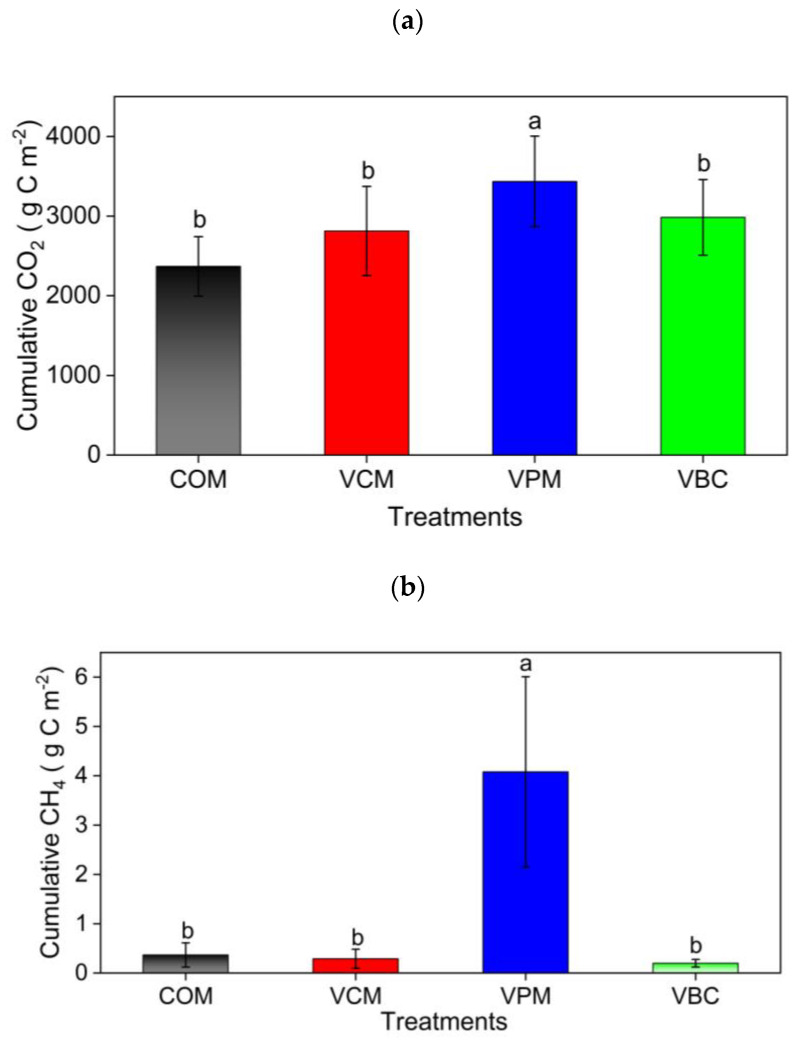

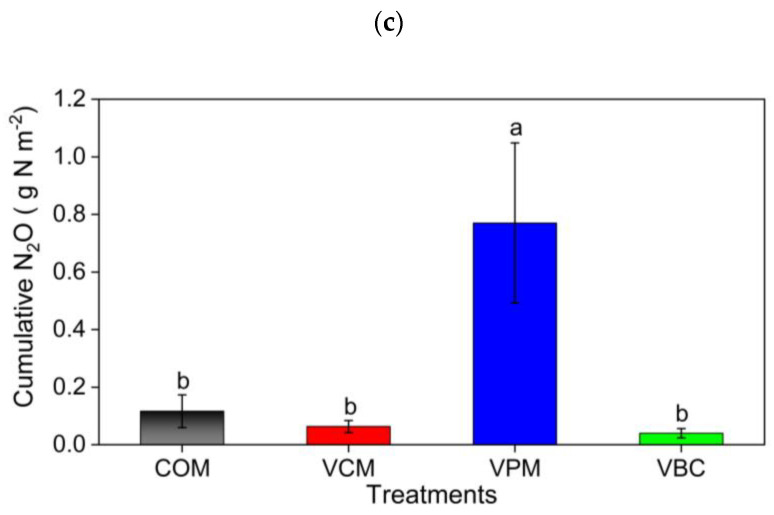
Cumulative carbon dioxide (**a**) methane (**b**), and nitrous oxide (**c**) in treatments for the whole experimental duration as an average of three replicates. Error bars represents standard errors. Different letter indicates significant differences among treatments (*p* < 0.05).

The cumulative CO_2_ emissions were ordered as VPM > VBC > VCM > COM (Figure 7a). For the changes in CH_4_ emissions, all treatments showed higher peaks in the first two weeks and then decreased in the remaining period (Figure 6b). VPM showed the highest emission rates throughout the observation period, especially in the first month. The cumulative CH_4_ emissions were, in decreasing order: VPM (4.07 g C m^−2^) > COM (0.36 g C m^−2^) > VCM (0.28 g C m^−2^) > VBC (0.19 g C m^−2^). The cumulative CH_4_ emission of VPM was significantly greater than that of other three treatments (*p* < 0.05) (Figure 7b).

N_2_O emissions in all treatments peaked in the first two weeks and decreased afterward except the elevated small peaks in VPM treatment in the last weeks (Figure 6c). N_2_O emissions of VPM were significantly greater than those of VCM, VBC, and COM (*p* < 0.05) (Figure 7c). 

### 3.5. Overall Evaluation of the Vermicomposting Practices

Overall, decreasing trends in GHG emissions were observed over time in all the treatments. The maximum emissions of all GHG occurred in the pre-composting period. Emissions of CO_2_, CH_4_, and N_2_O peaked in the first two weeks of the experiment for all the treatments. Fluctuations were observed soon after the mixing of each compost pile. It is believed that particularly temperature and substrate quality impact GHG emissions [30]. In this study, the colder December climate slowed decomposition during vermicomposting compared to our summer experiment (data not shown). The elevated N_2_O in the last weeks of the experiment is likely due to the stimulating effects of increasing temperature in March and April. The highest emissions of CO_2_, CH_4_, and N_2_O in pig manure treatment (VPM) might be due to the more degradable substrates with initially higher TOC and TN levels (Table 2).

Regarding the emission of gases among all the treatments (Table 3), GWP was calculated as the sum of the high warming potential two gases CH_4_ and N_2_O. The global warming potential of the GHG emissions was calculated to range from 0.37 to 7.82 g CO_2_-eq/kg.

## 4. Discussion

During vermicomposting, decreased pH values from initial to final stages occurred due to the decomposition of organic matter, humic acid, and ammonium ions. Decreased pH and EC could enhance plant growth [47]. It is believed that the shifting of pH toward acidity was due to nitrogen and phosphorus mineralization [48,49]. The changes in the EC during vermicomposting have been attributed to the utilization of soluble salts by microorganisms for biosynthesis and also to the absorption of soluble salts by earthworms [50]. 

Nutrient dynamics and the significant differences (*p* < 0.05) within treatments are shown in Table 2. This is likely due to the decomposition of organic materials by the earthworms-stimulated microbial community, resulting in a loss of CO_2_ and organic carbon [51]. The maximum decrease in TOC content was observed in VBC (26.43%). It is consistent with the conclusion that biochar might decay faster than other substrates, as it created suitable conditions for the efficient growth and development of juveniles. These results also verified earlier observations [19] that the higher decomposition rate of vermicompost is likely due enhanced microbial activities [19].

Excretory products by earthworms as their mucus secretions are attributed to the increase N content during vermicomposting as compared to COM. The higher TP increase in the VPM treatment may be due to the phosphorylation of organic matter during vermicomposting [52]. The increase in potassium level with the addition of biochar was in accordance with earlier studies [53,54,55,56]. Biochar has a high cation exchange potential, so that potassium and ammonium in biochar are available to plants for use [54]. The increased TK in the three treatments can be attributed to organic matter decomposition by earthworms that transformed insoluble TK to soluble TK [55]. As the acquisition of sufficient nitrogen for tissue production becomes problematic for worms, as the high C:N ratio is a critical factor that limits earthworm population [44], the declining values of C:N ratio in all treatments (with or without earthworms) supports potential usage in agriculture. A balanced C:N ratio is also important for plant nutrient availability [56]. Earthworms have been shown to enhance the fragmentation of large particles and provide better oxygenation in compost piles, which helped in the decomposition of carbon compound decomposition [20]. One possible reason for decreases in the C:N ratio during composting is microbial respiration and earthworm assimilation, which can cause a loss of CO_2_ and accumulation of nitrogen [57]. As the C:N ratio approaches 20, they become limiting for the earthworm population [58,59,60,61]. After 5 months, the C:N ratio of the four treatments in this study decreased to an acceptable level (Figure 3e, Table 2). Due to the higher initial carbon content in the VBC treatment, it is suggested that mixtures including biochar may need longer composting before field application. The C:P ratio results also confirmed the quick breakdown and mineralization of animal waste materials using earthworms [59]. Vermicomposting could potentially enhance the transformation of unavailable phosphorous into valuable forms of crops. The higher increase of phosphorous (Table 2) could be promoted by the phosphorylation process in pig manure [48]. 

The change in DOC could be due to their respiratory activities and to the earthworm-enhanced microbial utilization of available carbon. It is suggested that earthworms had a direct effect on the mineralization of organic compounds in this study. N is an essential building block of organic molecules, such as amino acids [44]; an increased mineralization of plant residues attained by the conversion of ammonium nitrogen (NH_4_-N) during vermicomposting was also reported [49,62,63]. Compared to the traditional compost, vermicomposting showed higher contents of nutrients with biochar amendment [60], which supported that vermicomposting using cow dung and biochar improved the nutrient supply in agriculture in this study. The significant increases of N, P, and K during composting in all the treatments could be good for crop growth, suggesting that the vermicompost could be used as a suitable organic fertilizer. The vermicomposting treatments showed a faster decomposition of waste materials with a greater decreasing trend of C:N ratios and TOC than traditional composting. However, for a comprehensive evaluation, the adverse effects of composting for its environmental pollution due to gas emissions need to be evaluated simultaneously.

Increased emissions during the early period may have occurred due to high NH_4_^+^ concentrations in raw composted materials, which decreased gradually during the observation period. Fluctuations in NH_3_ after a steep initial decrease correlated with the mixing of compost piles in all treatments with different time intervals for better aeration. The decreasing trend of NH_3_ loss in composting was the result of increased temperature, which could accelerate the degradation of the organic wastes, and this phenomenon is reported by Lv et al. [64]. The greater decline in C:N during vermicomposting observed in VCM and VBC might explain the lower NH_3_ emissions compared to VPM (Table 3; Figure 5b). Our results were similar to the effects of C:N ratios and earthworms on gas emissions during the vermicomposting studied [61]. The lower temperature during our experimental duration was attributed to differences in NH_3_ volatilizations or moisture conditions with different substrates having different C:N values impacted as well [65]. In terms of ammonia release, vermicomposting with cow manure and biochar was better than vermicompost with pig manure or traditional composting. The release of NH_3_ in the first week might be due to the inhibition of nitrifying bacteria by high temperature in the compost piles, preventing the conversion of ammonium nitrogen to NO_3_-N [64].

Normally, if the substrates are in a larger amount, then earthworms result in a higher decomposition of substance [30] in CO_2_ emissions. The burrowing activities of earthworms decreased the cumulative methane emissions. Koubová et al. [60] found that due to the high concentration of the methanogen Methanosaricina in cattle feces before composting, CH_4_ emission was lower in a VCM treatment than COM. 

The statistical analysis also revealed a positive relationship between the C:N ratio and CO_2_ emissions, which was comparable to the results [31]. As greenhouse gas emissions are linked to compost stability, a relationship between GHG and C:N ratio was established. Compared to VPM, the greater decrease in C:N during composting in VCM and VBC might cause lower CO_2_ emissions and CH_4_ emissions. Our N_2_O emissions results are similar to previous research showing that increased emissions in pig manure were due to the elevated threshold availability of nitrogen determined by earthworms [62]. Anaerobic denitrification by earthworm gut microbiota [63] might account for the decreased N_2_O emissions in VCM and VBC compared to COM. Furthermore, the different types of earthworms’ species and different substrates also made a difference in N_2_O emissions compared to COM treatment [31]. This overall phenomenon applied to all the gases, including NH_3_, CO_2_, CH_4_, and N_2_O. It might be attributed to the higher input of carbon by crop residue, which constituted 70% of the total mass in this study, but it could also be due to the faster degradation and enhanced mineralization of N [31,66].

In the vermicomposting processes, a transformation of organic wastes by earthworms enhanced the decomposition process and created a nutrient-rich fertilizer. Environmental impacts in the form of GHG emissions were lower for VCM and VBC as compared to VPM but higher than COM in all cases. The earthworms’ presence resulted in a better nutrient supply for crop growth and a good quality vermicompost, which is very useful for controlling NH_3_ volatilization and GHG emissions compared to the traditional method of composting. When compared to composting without earthworms, the vermicomposting treatments resulted in a much-improved nutrient status (Table 2).

Additionally, both vermicomposting with cow dung and pig manure were encouraged by previous studies [66]. This work on vermicomposting with biochar provided further proof that earthworms can survive and reproduce in biochar amendments for agricultural purposes. Based on N content, VCM showed the highest quality of vermicompost, while significantly, VPM and VBC also achieved higher nutrient status than COM. All in all, considering all the nutrient supplying and environmental factors, VCM and VBC were proven to be the most applicable for agriculture due to their relatively equal nutrient contents at the final stage (Table 2 and Table 3).

## 5. Conclusions

The nutrient conversion, ammonia volatilization, and GHG emissions were observed simultaneously and with consistently high frequency. The results showed that high nutrient contents and an overall low C:N ratio can be achieved in composting via vermicomposting. Notably, VPM consistently produced good quality compost through vermicompost as compared to COM; it was proven to be suitable for plant growth. Overall, this study clearly highlighted the importance of earthworms (*Eisenia fetida*) for recycling and reusing organic waste into bio-fertilizer by vermicomposting with manure with biochar as a suitable substrate. The goal was achieved with a field experiment conducted for the quick decomposition of organic materials (wastes) in winter to recovered important nutrients. This study estimated the NH_3_ volatilization and GHG emissions during vermicompost preparation process with different substrates. Further study about the functions of these vermicompost types, the cost analysis and microbial activities during vermicompost preparation, and then, its application to the field needs to be done to verify their quality. 

## Figures and Tables

**Table 1:** Physicochemical properties of raw materials of vermicomposting (mean ± SD, *n* = 3).

Raw Materials	TN (g/kg)	TOC (g/kg)	TP (g/kg)	TK (g/kg)	C:N Ratio	pH	EC (µS|cm)
Crop residues (Maize)	10.72 ± 1.0	391.6 ± 8.1	1.23 ± 1.0	12.59 ± 0.6	36.5 ± 2.3	6.4 ± 0.0	3.6 ± 0.4
Pig manure	18.27 ± 0.2	334.9 ± 9.7	5.89 ± 3.7	11.14 ± 2.3	18.32 ± 0.6	8.3 ± 0.0	2.8 ± 0.0
Cow dung	25.48 ± 1.1	281.9 ± 7.8	7.78 ± 1.5	6.64 ± 0.9	11.06 ± 0.6	6.8 ± 0.1	4.4 ± 1.8
Biochar	10.4 ± 0.8	446.8 ± 15.1	1.95 ± 26	32.6 ± 7.2	42.95 ± 3.7	8.8 ± 0.1	13.7 ± 0.5

**Table 2:** Nutrient (g/kg) change during vermicomposting.

Treatments	COM			VCM			VPM			VBC		
	Initial	Final	Increase %	Initial	Final	Increase %	Initial	Final	Increase %	Initial	Final	Increase %
TOC	341.1 ± 20.8	268.2 ± 7.4 b	*−21.34	337.3 ± 44.5	261.0 ± 42.1 b	*−22.62	353.6 ± 37.9	261.5 ± 18.0 c	*−26.05	398.4 ± 7.7	293.1 ± 24.0 a	*−26.43
TN	12.1 ± 0.9	16.2 ± 0.9 a	33.88	12.3 ± 1.17	20.4 ± 1.2 a	65.85	12.8 ± 1.3	16.3 ± 0.7 a	27.34	10.4 ± 0.7	13.8 ± 0.1 b	31.73
TP	2.82 ± 0.18	3.52 ± 0.4 b	24.82	2.34 ± 0.55	3.78 ± 0.32 b	61.54	1.48 ± 0.30	6.13 ± 0.48 a	314.19	1.24 ± 0.65	1.94 ± 0.30 c	56.45
TK	10.34 ± 0.60	10.30 ± 1.5 b	*−0.39	9.00 ± 0.92	10.98 ± 0.44 b	22.00	7.37 ± 1.21	14.25 ± 0.53 b	93.35	13.75 ± 1.28	18.64 ± 1.02 a	35.56
C:N ratio	28.10 ± 2.32	16.57 ± 2.12	*−41.03	27.52 ± 3.77	12.78 ± 33.08	*−53.56	27.73 ± 3.32	16.01 ± 70.43	*−42.26	38.49 ± 5.94	21.34 ± 3.44	*−44.56
C:P ratio	12.10	7.62	−36.98	14.41	6.90	*−52.10	23.89	4.27	*−82.14	32.13	15.11	*−52.98
N:P ratio	0.43	0.46	7.26	0.53	0.54	2.67	0.86	0.27	*−69.25	0.84	0.71	*−15.80

**Note:** Values are shown as mean ± standard deviation (*n* = 3). Asterisks (*) indicate a greater decrease in ratios. Letters (a, b, c) indicate significant differences within a given row (*p* < 0.05).

**Table 3:** Total greenhouse gases (GHG) emissions and global warming potential (GWP) in all treatments.

Treatments	GHGs Emissions Equivalent (g CO_2_-eq/kg)			
	CO_2_	CH_4_	N_2_O	GWP
COM	130.4	0.007	0.002	0.83
VCM	154.9	0.005	0.001	0.46
VPM	189.1	0.081	0.017	7.82
VBC	164.2	0.003	0.000	0.37

## Data Availability

The data availability on request from corresponding author.
